# Down-Shifting and Anti-Reflection Effect of CsPbBr_3_ Quantum Dots/Multicrystalline Silicon Hybrid Structures for Enhanced Photovoltaic Properties

**DOI:** 10.3390/nano10040775

**Published:** 2020-04-17

**Authors:** Yunqing Cao, Dong Wu, Ping Zhu, Dan Shan, Xianghua Zeng, Jun Xu

**Affiliations:** 1 College of Physical Science and Technology, Yangzhou University, Yangzhou 225009, China; wudong_19931125@163.com (D.W.); zhuping1397951458@163.com (P.Z.); xhzeng@yzu.edu.cn (X.Z.); 2 National Laboratory of Solid State Microstructures and School of Electronic Science and Engineering and Collaborative Innovation Center of Advanced Microstructures, Nanjing University, Nanjing 210093, China; shandnju@gmail.com (D.S.); junxu@nju.edu.cn (J.X.); 3 School of Electronic and Information Engineering, Yangzhou Polytechnic Institute, Yangzhou 225127, China

**Keywords:** caesium lead bromide perovskite quantum dots (CsPbBr_3_ QDs), multicrystalline Si (mc-Si), solar cell, down-shifting effect, anti-reflection property

## Abstract

Over the past couple of decades, extensive research has been conducted on silicon (Si) based solar cells, whose power conversion efficiency (PCE) still has limitations because of a mismatched solar spectrum. Recently, a down-shifting effect has provided a new way to improve cell performances by converting ultraviolet (UV) photons to visible light. In this work, caesium lead bromide perovskite quantum dots (CsPbBr_3_ QDs) are synthesized with a uniform size of 10 nm. Exhibiting strong absorption of near UV light and intense photoluminescence (PL) peak at 515 nm, CsPbBr_3_ QDs show a potential application of the down-shifting effect. CsPbBr_3_ QDs/multicrystalline silicon (mc-Si) hybrid structured solar cells are fabricated and systematically studied. Compared with mc-Si solar cells, CsPbBr_3_ QDs/mc-Si solar cells have obvious improvement in external quantum efficiency (EQE) within the wavelength ranges of both 300 to 500 nm and 700 to 1100 nm, which can be attributed to the down-shifting effect and the anti-reflection property of CsPbBr_3_ QDs through the formation of CsPbBr_3_ QDs/mc-Si structures. Furthermore, a detailed discussion of contact resistance and interface defects is provided. As a result, the coated CsPbBr_3_ QDs are optimized to be two layers and the solar cell exhibits a highest PCE of 14.52%.

## 1. Introduction

For the past few years, silicon (Si) based solar cells have become the most commonly-used materials of photovoltaic devices because of its abundant and non-polluting properties with a mature production process [1,2,3]. By virtue of its high efficiency and low cost, multicrystalline Si (mc-Si) solar cells are produced most extensively among various solar cells [4]. However, crystalline Si solar cells are limited in power conversion efficiency (PCE), as high-energy photons cannot fully be utilized and photons whose energy is inferior to the bandgap of Si have transmission loss [5]. In order to obtain better spectral response, one possible solution is to use luminescent materials converting ultraviolet (UV) photons to visible light by means of a down-shifting effect [6,7]. Recently, multiple reports have demonstrated that the down-shifting mechanism of nanomaterials can improve the PCE of solar cells [8,9,10,11,12]. For example, van Sark et al. calculated in theory that Cd-based quantum dots (QDs), which had an emission at 603 nm, led to an increase of around 10% in the short-circuit current of mc-Si solar cells [13]. Pi et al. fabricated Si QDs on the surface of mc-Si solar cells via the inkjet printing method and found that solar cell exhibited a relative rise of 2% in PCE because of better spectral response within a short wavelength range of 300 nm to 400 nm [14]. On one hand, however, conventional CdS or CdSe QDs are faced with the problems of severe aggregation and photoluminescence (PL) quenching in the process of film fabrication [15]. On the other hand, Si QDs fail to achieve high PL quantum yield (QY) due to their indirect Si bandgap [16,17,18].

With a potential application in light emitting diodes [19,20,21], lasers [22,23,24], photodetectors [25,26,27] and other optoelectronic devices, all inorganic lead halide perovskite QDs (IPQDs) could become alternative materials for the down-shifting effect and photovoltaic applications due to their low cost synthesis method, long-time stability, high optical absorption coefficient, as well as controllable and high intensity PL [28,29,30]. Compared with a relatively low PL QY (<50%) in Si QDs [31,32], IPQDs have PL QYs of 80%, 95%, 70%, for red, green, and blue emissions [33]. It has been reported that the spectral response with near UV light range of 300–390 nm of a Cu(In,Ga)Se_2_ (CIGS) thin film solar cell was improved by taking advantage of the down-shifting effect of IPQDs [34]. In the present work, a colloidal approach is introduced to synthesize caesium lead bromide perovskite quantum dots (CsPbBr_3_ QDs) that are cubic shaped with a mean size of 10 nm and whose room temperature PL peak is observed at 515 nm. The colloidal CsPbBr_3_ QDs solution is then spin-coated onto the surface of commercially produced mc-Si solar cells in order to obtain CsPbBr_3_ QDs/mc-Si hybrid structured solar cells. It is found that CsPbBr_3_ QDs/mc-Si hybrid structured solar cells have an increase in external quantum efficiency (EQE) within the wavelength ranges of both 300 to 500 nm and 700 to 1100 nm compared with solar cells without CsPbBr_3_ QDs, demonstrating that the photovoltaic performances of solar cells can be improved by the down-shifting effect and the anti-reflection property of CsPbBr_3_ QDs/mc-Si hybrid structures.

## 2. Materials and Methods

CsPbBr_3_ QDs were synthesized with a colloidal approach. First, Cs_2_CO_3_ (99.9%, 2.5 mmol, Sigma-Aldrich, St. Louis, MO, USA) was added to 40 mL of octadecene (ODE, 90%, Acros, Geel, Belgium) with 2.5 mL of oleic acid (OA, 90%, Sigma-Aldrich) at 130 °C under N_2_ atmosphere for 30 min. The solution after reaction was naturally cooled to ambient temperature to obtain the Cs-oleate solution. Second, PbBr_2_ (99.999%, 0.188 mmol, Sigma-Aldrich), 5 mL of ODE, 0.5 mL of OA, and 0.5 mL of oleylamine (OLA, 90%, Acros) were mixed and dried at 120 °C under vacuum for 60 min. After complete dissolution of PbBr_2_, the solution saw a rise of 150 °C in temperature under N_2_ atmosphere and a quick injection of 0.4 mL of the prepared Cs-oleate solution. The mixture after reaction was placed at this temperature for 5 s and cooled by an ice-water bath to ambient temperature. Finally, the reaction solution was used to purify CsPbBr_3_ QDs by centrifugation at 12,000 rpm for 10 min and then re-dispersed in n-hexane (99%, Sigma-Aldrich) to obtain a long-time stable colloidal solution.

The microstructures of CsPbBr_3_ QDs were characterized by means of transmission electron microscopy (TEM) and X-ray diffraction (XRD) (MXP-III, Bruker, Inc., Leipzig, Germany). The diluted and highly dispersed CsPbBr_3_ QDs solution was dropped onto a carbon-coated Cu grid and dried at room temperature. TEM images were performed by Tecnai G2 operated at 200 kV. A UV-3600 spectrophotometer produced by Shimadzu was applied to measure the optical absorbance of CsPbBr_3_ QDs. Equipped with a synapse photomultiplier tube (PMT) detector, a system made by HORIBA Jobin Yvon was used to measure PL spectra. The absorbance and PL spectra were measured at room temperature in a fused silica cuvette using diluted solution of CsPbBr_3_ QDs in n-hexane.

In this work, commercially produced mc-Si solar cells with texturized surface were fabricated by Hareonsolar (Wuxi, China), including acid texturization, high temperature diffusion, SiN*_x_* film deposition, and metal grid screen-printing. The mc-Si substrate was p-type (1~3 Ω·cm) with a thickness of 200 ± 20 μm and the texturized surface had an average roughness of 0.8~1.6 μm. The surface of mc-Si solar cells was spin-coated with the CsPbBr_3_ QDs/n-hexane solution at 2000 rpm for 1 min in a glovebox. CsPbBr_3_ QDs have a concentration of 5 mg/mL and mc-Si solar cells have an active area of 4 cm^2^. Here, layer-by-layer spin-coating process was used to fabricate CsPbBr_3_ QDs/mc-Si hybrid structured solar cells. The layer number of CsPbBr_3_ QDs varied from one to four. A 610C electrometer made by Keithley (Cleveland, OH, USA) was utilized to measure the current density-voltage (*J-V*) characteristics of solar cells under the AM 1.5 (100 mW/cm^2^) illumination. A QEX10 quantum efficiency/spectral response measurement system produced by PV Measurements (Point Roberts, WA, USA) was adopted to measure the EQE spectra of mc-Si solar cells inclusive and exclusive of CsPbBr_3_ QDs within the spectral range of 300 to 1100 nm. A standard Si solar cell was used for calibrating both *J-V* and EQE measurements. Hall Effect was measured at room temperature by LakeShore 8400 with the use of a coplanar van der Pauw (VDP) geometry and films with vacuum-evaporated Al electrodes. A liquid He-cooled spectrometer produced by Bruker EMX (Karlsruhe, Germany) was utilized to obtain low-temperature X-band electron spin resonance (ESR) spectra CsPbBr_3_ QD films.

## 3. Results

### 3.1. Structural Characterizations of CsPbBr_3_ QDs

**Figure 1:**
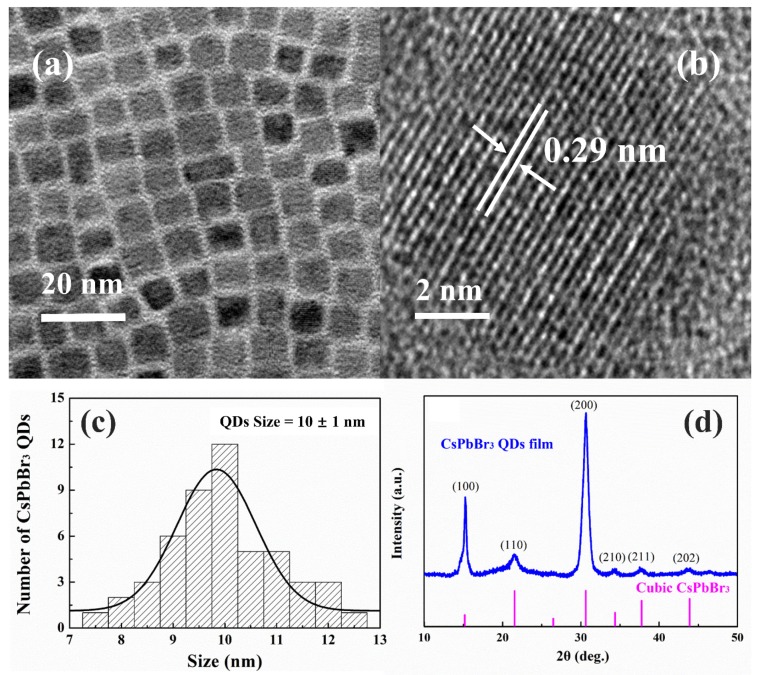
(**a**) TEM and (**b**) HRTEM images of CsPbBr_3_ QDs. (**c**) Histogram for the size distribution of the CsPbBr_3_ QDs. (**d**) XRD spectrum of CsPbBr_3_ QDs film.

Figure 1a shows that CsPbBr_3_ QDs prepared in the current experiment present cubic shapes with a uniform size. The high-resolution TEM (HRTEM) image of one CsPbBr_3_ QD is presented in Figure 1b. The prepared CsPbBr_3_ QDs have a crystalline interplanar spacing of 0.29 nm relative to the (200) crystalline planes of cubic CsPbBr_3_. The distribution of CsPbBr_3_ QDs sizes is demonstrated in Figure 1c. The quantum confinement effect of CsPbBr_3_ QDs is expectable since their mean size (about 10 nm) approaches the Bohr diameter (7 nm) that was predicted by the Wannier-Mott excitons of bulk CsPbBr_3_ perovskites [35]. The pattern of XRD presented in Figure 1d further confirms the cubic crystalline structures of prepared CsPbBr_3_ QDs. Characteristic diffraction peaks at 15.2°, 21.5°, 30.6°, 34.3°, 37.7°, and 43.8° can be allocated to diffractions from (100), (110), (200), (210), (211), and (202) crystalline planes of cubic CsPbBr_3_ (JCPDS Card No. 54-0752), respectively, which is aligned with TEM results.

### 3.2. Optical Properties of CsPbBr_3_ QDs

**Figure 2:**
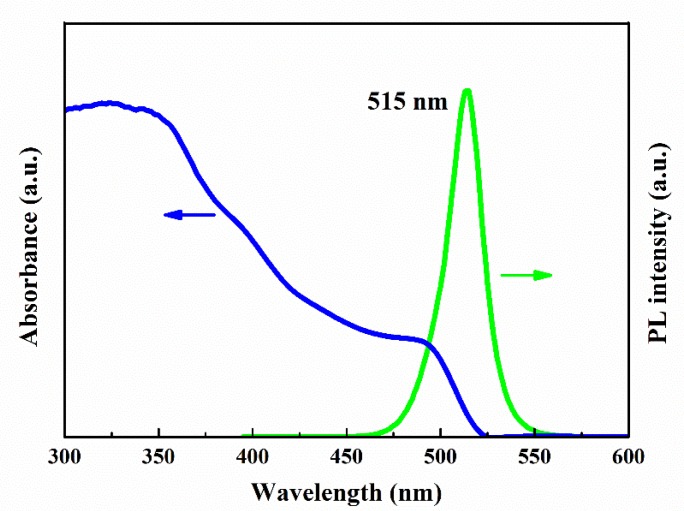
Optical absorption (blue line) and PL (green line) spectra of CsPbBr_3_ QDs.

It can be seen in Figure 2 that CsPbBr_3_ QDs have quite high absorbance in the region of short wavelength and the absorption edge locates at 520 nm (2.38 eV), suggesting that the blue-shift of the bandgap corresponds to bulk CsPbBr_3_ with 2.25 eV [36], which accords with the quantum confinement effect. In addition, the PL spectrum of CsPbBr_3_ QDs excited by a He-Cd laser of 325 nm is also presented in Figure 2, showing an intense peak at 515 nm with width as narrow as 20 nm at half height. The observed high intensity and color purity PL can be attributed to the uniform size distribution of CsPbBr_3_ QDs, as identified by TEM measurements. Moreover, the PL QY is measured to be as high as 80%, by using standard fluorescence dye as a reference. It is demonstrated that CsPbBr_3_ QDs can strongly absorb the near UV photons and then efficiently emit visible light, indicating their suitable down-shifting applications.

### 3.3. Photovoltaic Properties of CsPbBr_3_ QDs/mc-Si Hybrid Structured Solar Cells

**Figure 3:**
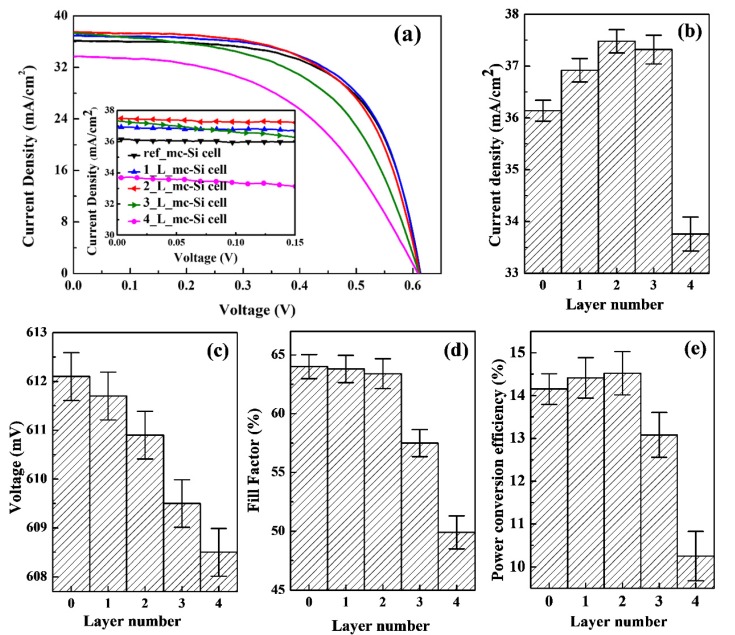
(**a**) AM 1.5G illuminated *J-V* curves of mc-Si solar cells inclusive and exclusive of CsPbBr_3_ QDs layers. (**b**) Short circuit current density (*J_sc_*), (**c**) open circuit voltage (*V_oc_*), (**d**) fill factor (FF), and (**e**) power conversion efficiency (PCE) of solar cells.

CsPbBr_3_ QDs/mc-Si hybrid structured solar cells were fabricated after spin-coating the colloidal CsPbBr_3_ QDs solution onto the surface of mc-Si solar cells. Measurements were carried out for the *J-V* characteristics of solar cells under the AM 1.5 illumination. Figure 3a shows the illuminated *J-V* curves of mc-Si solar cells inclusive and exclusive of CsPbBr_3_ QDs. The photovoltaic parameters, including short circuit current density (*J_sc_*), open circuit voltage (*V_oc_*), fill factor (FF), and PCE, are summarized as shown in Figure 3b–e. To simplify statement, the CsPbBr_3_ QDs/mc-Si hybrid structured solar cells are named as 1_L_mc-Si cell, 2_L_mc-Si cell, 3_L_mc-Si cell, and 4_L_mc-Si cell corresponding to the layers of spin-coated CsPbBr_3_ QDs. Besides, the mc-Si solar cell without CsPbBr_3_ QDs is named as ref_mc-Si cell for reference.

**Figure 4:**
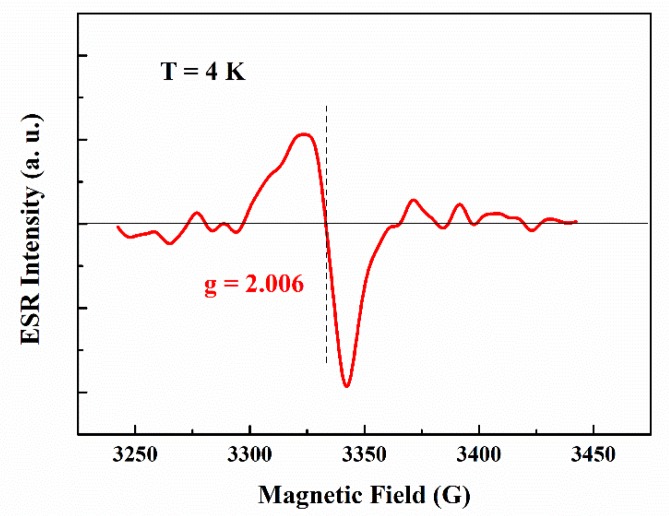
Low temperature ESR spectrum of the CsPbBr_3_ QDs/mc-Si structure.

In general, cell performances strongly depend on the layer number of CsPbBr_3_ QDs. As shown in Figure 3b, CsPbBr_3_ QDs/mc-Si hybrid structured solar cells have higher *J_sc_* compared with ref_mc-Si solar cell when the layer number of CsPbBr_3_ QDs is one to three. In particular, *J_sc_* increases from 36.14 mA/cm^2^ to 37.48 mA/cm^2^ when the layer number is two, which is considered to result from better spectral utilization. However, the *J_sc_* of 4_L_mc-Si cell drops to 33.76 mA/cm^2^, even below that of mc-Si solar cell without CsPbBr_3_ QDs. In principle, the significant reduction of *J_sc_* can be explained by the following two reasons. The first reason should be the induced contact resistance in CsPbBr_3_ QDs layers. As we know, undoped CsPbBr_3_ QDs have poor electrical conductivity. Through the room temperature Hall Effect measurement, the CsPbBr_3_ QDs film has a dark conductivity of about 3.6 × 10^−7^ S/cm, which is very low in the order of magnitude according to the previous reports of metal halide perovskite materials [37,38,39]. In this study, *J_sc_* drops off due to the increase of contact resistance, which deteriorates the carrier collection efficiency. The other reason is the defect in hybrid structures which acts as a non-radiative recombination center and results in thermal energy loss. In order to confirm the existence and study the behavior of defects in hybrid structures, a liquid He-cooled spectrometer of Bruker EMX 10/12+ whose center field is 3342.5 G is used to obtain the low-temperature X-band ESR spectrum of CsPbBr_3_ QDs/mc-Si structure, as shown in Figure 4. The X-band ESR spectrum at 4 K shows an ESR signal with g = 2.006. In previous works, this signal was usually observed in disordered materials and corresponding to the dangling bond defects derived from the chemical synthesis and spin-coating process, and poor adhesion of coating films on substrates [40,41,42]. It is worth noting that no ESR signal can be detected in the ref_mc-Si solar cell. As a result, the ESR signal here results from the lone-pair electrons (like dangling bonds defects) on the surface and interfaces of CsPbBr_3_ QDs layers. The density of induced surface and interface defects increases with the increasing layer number of CsPbBr_3_ QDs, indicating a decline in the *J_sc_* of solar cells because of the enhanced carrier recombination.

As shown in Figure 3c, the change of *V_oc_* with the layer number of CsPbBr_3_ QDs tends to be linear. When the layer number increases, *V_oc_* decreases slightly from 612.1 to 608.5 mV because of the surface and interface defects induced by CsPbBr_3_ QDs layers. As shown in Figure 3d, FF declines obviously when the layer number increases to four, which may be owing to an increase in series resistance (*R_s_*). A negative correlation is found between FF and *R_s_*. Equation (1) below displays the relationship between the voltage and current density of a single-diode model:

(1) $$J=J_{0}\left(exp\left(\frac{q\left(V-R_{s}J\right)}{nk_{B}T}\right)-1\right)+\frac{V-R_{s}J}{R_{sh}}-J_{p}$$

wherein, *J_0_* and *J_p_* are saturation current density and photocurrent density, respectively, *R_s_* represents series resistance, *R_sh_* represents shunt resistance, *n* refers to ideality factor, *q* is electron charge, *k_B_* is Boltzmann constant and *T* is temperature. The *R_s_* values of ref_mc-Si and 4_L_mc-Si cells extracted by a fit to the illuminated *J-V* curves are 1.2 and 2.8 Ω, respectively. As discussed before, contact resistance increases with the increasing layer number of CsPbBr_3_ QDs, which in turn give rise to an obvious decline in FF. Finally, the optimal mc-Si solar cell coated with two layers of CsPbBr_3_ QDs leads to the best PCE of 14.52%, as shown in Figure 3e.

**Figure 5:**
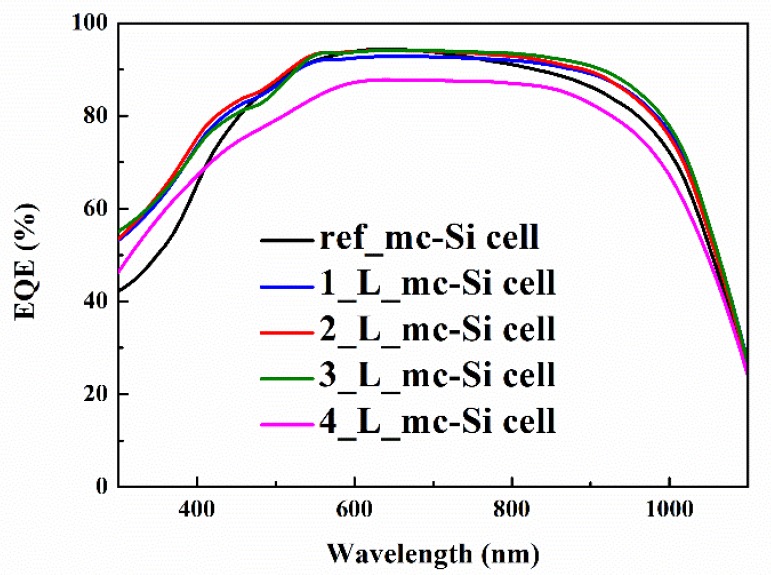
External quantum efficiency (EQE) spectra of mc-Si solar cells inclusive and exclusive of CsPbBr_3_ QDs layers.

The EQE spectra of mc-Si solar cells inclusive and exclusive of CsPbBr_3_ QDs layers were measured to further study the down-shifting mechanism of CsPbBr_3_ QDs/mc-Si hybrid structured solar cells. As observed in Figure 5, the EQE of CsPbBr_3_ QDs/mc-Si hybrid structured solar cells has a great increase within the spectral range of 300 to 500 nm when the layer number of CsPbBr_3_ QDs is 1 to 3, which should be attributed to the formation of CsPbBr_3_ QDs, as discussed before. It suggests that CsPbBr_3_ QDs are capable of absorbing the near UV light and emitting photons in the region of visible light, which could enhance the spectral response in the region of short wavelength through the re-absorption of mc-Si substrates. When the layer number increases to 4, nevertheless, EQE decreases obviously almost in the whole spectral range, even below that of ref_mc-Si cell, which agrees with the *J-V* results. Another interesting finding is that the EQE of CsPbBr_3_ QDs/mc-Si hybrid structured solar cells also increases within the spectral range of 700 to 1100 nm, which will be discussed below. Based on EQE results, *J_sc_* contributed from the spectral response of solar cells in different wavelength ranges can be calculated according to Equation (2) as follows:

(2) $$J_{sc}=\int_{\lambda_{1}}^{\lambda_{2}}\frac{F\left(\lambda \right)\cdot EQE\left(\lambda \right)}{E\left(\lambda \right)}d\lambda$$

wherein, F(λ) and E(λ) are incident light flux and energy of photons with the wavelength of λ, respectively. Compared with ref_mc-Si cell, the *J_sc_* of 2_L_mc-Si cell contributed from spectral response within spectral range of 300 to 500 nm and 700 to 1100 nm increases from 5.98 mA/cm^2^ to 6.79 mA/cm^2^ and 14.15 mA/cm^2^ to 14.52 mA/cm^2^, respectively. As reported by the work of Pi et al., the anti-reflection of prepared porous Si QDs films improved the efficiency of crystalline Si (c-Si) solar cells [43]. In this study, the reflection characteristics of mc-Si solar cells inclusive and exclusive of CsPbBr_3_ QDs layers must be taken into consideration.

**Figure 6:**
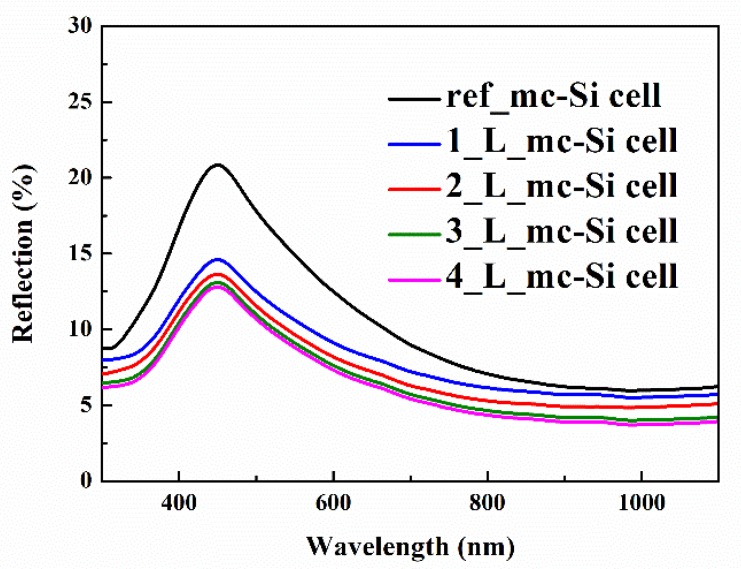
Optical reflection spectra of mc-Si solar cells inclusive and exclusive of CsPbBr_3_ QDs layers.

Figure 6 shows the optical reflection (R) spectra of mc-Si solar cells and CsPbBr_3_ QDs/mc-Si hybrid structured solar cells. Obviously, CsPbBr_3_ QDs layers lead to the decrease of reflection within the spectral ranges of both 300 to 500 nm and 700 to 1100 nm. The reduction of reflection in long wavelength region is mainly induced by the nanostructure of CsPbBr_3_ QDs layers. Meanwhile, a greater decline in reflection in short wavelength region should be ascribed to the anti-reflection property of nanostructures and the absorption of CsPbBr_3_ QDs themselves, as shown in Figure 2.

**Figure 7:**
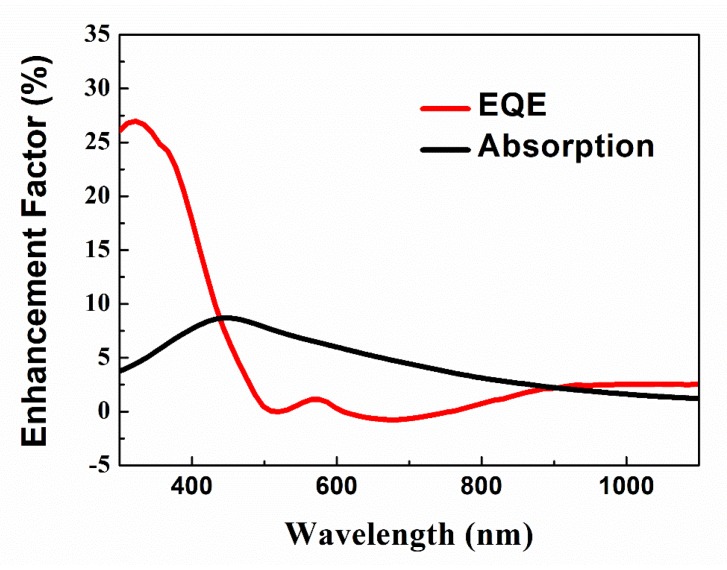
Enhancement factor (EF) of mc-Si solar cell coated with two layers of CsPbBr_3_ QDs as a function of wavelength.

In order to evaluate the contributions from the down-shifting and anti-reflection effect, an enhancement factor (EF) is defined for both the EQE and absorption results:

(3) $$EF_{EQE}=\frac{EQE_{with\;QDs}-EQE_{without\;QDs}}{EQE_{without\;QDs}}$$

(4) $$EF_{A}=\frac{A_{with\;QDs}-A_{without\;QDs}}{A_{without\;QDs}}$$

wherein, A is the optical absorption of hybrid structures, which can be deduced by A = 1 − R. Figure 7 shows the EF of 2_L_mc-Si cell as a function of wavelength. It can be clearly seen that the decrease of reflection (which means the enhancement of absorption) improves EQE within the wavelength range of 700 to 1100 nm. However, in wavelength range of 300 to 450 nm, the anti-reflection effect cannot overlap with all the enhancement of EQE result. For example, at 320 nm, the EQE enhancement is 27% while the absorption enhancement is only 4%, which indicates that the down-shifting effect of CsPbBr_3_ QDs dominates the improving spectral response in the short wavelength region. In contrast, at 480 nm, the CsPbBr_3_ QDs/mc-Si structures show the strongest anti-reflection property. The enhancements of EQE and absorption are almost the same. Hence, the EQE enhancement is mainly due to the anti-reflection of hybrid structures. In a word, we conclude that the improved photovoltaic performances can be attributed both to the down-shifting effect and the anti-reflection property of CsPbBr_3_ QDs by forming CsPbBr_3_ QDs/mc-Si hybrid structures. Furthermore, for future photovoltaic application, in order to avoid the environmental pollution caused by lead, a protected layer will be introduced into the hybrid structures to reduce the lead leakage [44,45].

## 4. Conclusions

In conclusion, CsPbBr_3_ QDs are fabricated with a colloidal synthesis approach. TEM and XRD measurements reveal that cubic CsPbBr_3_ QDs are formed with an average size of 10 nm. It is observed that CsPbBr_3_ QDs can strongly absorb the near UV light and present a room temperature PL peak at 515 nm, suggesting their typical down-shifting mechanism. Moreover, CsPbBr_3_ QDs/mc-Si hybrid structured solar cells containing CsPbBr_3_ QDs of different layers are fabricated. According to the findings, the EQE of CsPbBr_3_ QDs/mc-Si hybrid structured solar cells is increased within the wavelength ranges of both 300 to 500 nm and 700 to 1100 nm compared with mc-Si solar cells without CsPbBr_3_ QDs, which should be attributed to the broadband anti-reflection characteristics of nanostructures and the additionally improved down-shifting effect of CsPbBr_3_ QDs. However, the higher contact resistance and density of surface and interface defects resulting from the increasing layer number of CsPbBr_3_ QDs deteriorate cell performances due to the reduction of carrier collection efficiency. As a result, the optimal mc-Si solar cell coated with two layers of CsPbBr_3_ QDs contributes to achieving the best PCE of 14.52%. It is worth noting that all inorganic CsPbBr_3_ QDs have better stability than the organic-inorganic (MAPbBr_3_) perovskite materials. Our experimental results indicate a promising way to exploit CsPbBr_3_ QDs for future photovoltaic devices.
